# Carbon dioxide dialysis in a swine model utilizing systemic and regional anticoagulation

**DOI:** 10.1186/s40635-016-0076-3

**Published:** 2016-01-16

**Authors:** A. S. Sharma, P. W. Weerwind, O. Bekers, E. M. Wouters, J. G. Maessen

**Affiliations:** Department of Cardiothoracic Surgery, CARIM, Maastricht University Medical Center, PO box 5800, 6202 AZ Maastricht, the Netherlands; Department of Clinical Chemistry, Maastricht University Medical Center, Maastricht, the Netherlands; Department of Respiratory Medicine, NUTRIM, Maastricht University Medical Center, Maastricht, the Netherlands

**Keywords:** Acute-on-chronic hypercapnic respiratory failure, Low-flow CO_2_ removal, Regional anticoagulation, Systemic anticoagulation, Animal model

## Abstract

**Background:**

Extracorporeal carbon dioxide removal (ECCO_2_R) has been gaining interest to potentially facilitate gas transfer and equilibrate mild to moderate hypercapnic acidosis, when standard therapy with non-invasive ventilation is deemed refractory. However, concern regarding the effectiveness of low-flow CO_2_ removal remains. Additionally, the prospect to steadily reduce hypercapnia via low-flow ECCO_2_R technique is limited, especially with regional anticoagulation which potentially reduces the risk of bleeding. Therefore, an in vivo study was conducted to determine the efficacy of CO_2_ removal through a modified renal dialysis unit during the carbon dioxide dialysis study using systemic and regional anticoagulation.

**Methods:**

The acute study was conducted for 14 h in landrace pigs (51 ± 3 kg). CO_2_ removal using a diffusion membrane oxygenator substituting the hemoconcentrator was provided for 6 h. Blood and gas (100 % O_2_) flows were set at 200 and 5 L/min, respectively. Anticoagulation was achieved by systemic heparinization (*n* = 7) or regional trisodium citrate 4 % (*n* = 7).

**Results:**

The CO_2_ transfer was highest during the initial hour and ranged from 45 to 35 mL/min, achieving near eucapnic values. Regional and systemic anticoagulation were both effective in decreasing arterial pCO_2_ (from 8.9 ± 1.3 kPa to 5.6 ± 0.8 kPa and from 8.6 ± 1.0 kPa to 6.3 ± 0.7 kPa, *p* < 0.05 for both groups, respectively). Furthermore, pH improved (from 7.32 ± 0.08 to 7.47 ± 0.07 and from 7.37 ± 0.04 to 7.49 ± 0.01, *p* < 0.05) for both regional and systemic anticoagulation groups, respectively. Upon ceasing CO_2_ dialysis, hypercapnia ensued. The liver and kidney function test results were normal, and scanning electron microscopy analysis revealed only some cellular and fibrin adhesion on the oxygenator fibre in the heparin group.

**Conclusions:**

CO_2_ dialysis utilizing either regional or systemic anticoagulation showed to be safe and effective in steady transfer of CO_2_ and consequently optimizing pH.

## Background

Chronic obstructive pulmonary disease (COPD) patients presenting with mild to moderate hypercapnic respiratory acidosis are often supported by non-invasive ventilation (NIV) to facilitate gas transfer and prevent progression into severe acidosis [1]. In about 25 to 50 % of these patients, NIV may not be effective in improving the respiratory mechanics with a consequent need for invasive ventilation [2]. Hence, alternative approaches to facilitate gas transfer have been sought, including the application of extracorporeal carbon dioxide removal (ECCO_2_R) technique [3]. ECCO_2_R technique primarily supports decarboxylation and thereby improves respiratory acidosis, finding its application relevant in reversible type 2 respiratory failure situations.

CO_2_ is primarily transported in a dissolved state in plasma and can be extracted effectively through a membrane lung when connected to high sweep gas flows [4, 5]. Other determinants that influence gas transfer are membrane surface area, partial pressure of CO_2_ (pCO_2_) concentration before the membrane lung, and even the type of membrane [5–7]. Terragni et al. describe that ECCO_2_R application at sweep gas flows of 6 to 8 L/min and blood flows of approximately 500 mL/min has shown to extract approximately 80 mL of CO_2_/min, which accounts for 40 % of the calculated CO_2_ production [3]. While Burki et al. in a pilot study reported that with ECCO_2_R application at low blood flow (approximately 500 mL/min), a subset of patients with underlying COPD could be managed without invasive mechanical ventilation [8]. Although, these described techniques require the use of specially designed cannula or even devices [9–11] that could increase the healthcare costs. Since COPD is a growing concern world-wide, adapting a pre-existing renal dialysis unit and providing CO_2_ removal at even lower blood flows through the modified system could potentially support patients over an extended period of time to avert rapid correction of the acid-base balance in mild to moderate acidosis, when NIV is contradicted or proves to be insufficient.

Furthermore, to prevent clotting of the extracorporeal circuit, heparin is administered as a systemic anticoagulant [8]. However, heparin induces bleeding and has been reported up to 40 % of patients placed on veno-venous extracorporeal support [12, 13]. Thus, this concern also extends to the application of ECCO_2_R technique. Alternatively, regional anticoagulation likely circumvents heparin-induced bleeding and seems to be an attractive option [14]. Regional anticoagulation with trisodium citrate has been extensively used to maintain circuit patency with safer and simple protocols in renal replacement therapy [15, 16]. Moreover, exogenously administered citrate metabolizes to sodium bicarbonate [17], which might be beneficial in acute respiratory acidosis. However, this chemical buffering effect in low-flow ECCO_2_R systems has not been verified.

We hypothesize that implementing dialysis flows and opting for regional anticoagulation with trisodium citrate might benefit patients with hypercapnic respiratory failure. Therefore, in a swine model, the efficacy of CO_2_ removal at a dialysis flow rate using regional trisodium citrate anticoagulant and also systemic anticoagulant was determined utilizing a modified renal dialysis unit.

## Methods

This in vivo study was conducted at the Department of Cardiothoracic Surgery, Experimental Laboratory, Maastricht University, the Netherlands. The study was approved by the local animal investigation committee of the Maastricht University. Animal handling was in accordance with the guide and use of laboratory animals (published by the National Institute of Health, USA).

### Overview of study

Fourteen healthy domestic landrace pigs weighing 51 ± 3 kg were studied. The animals were divided into two groups based on either systemic anticoagulation with heparin (group 1, *n* = 7) or regional anticoagulation using 4 % trisodium citrate (group 2, *n* = 7).

Each experiment was conducted over a duration of 14 h, divided into four subsequent phases: stabilization phase (0–60 min), hypercapnic phase (60–360 min), intervention phase (360–720 min), and post-intervention phase (720–840 min).

### Animal preparation

The animals were sedated with a mixture of intramuscular administrated ketamine (10 mg/kg), midazolam (0.5 mg/kg), and atropine (0.04 mg/kg). Induction of anaesthesia and analgesia was achieved by an intravenous injection of thiopental sodium (5 mg/kg), midazolam (0.5 mg/kg), and sufentanyl citrate (6 μg/kg). Following endotracheal intubation, mechanical ventilation (Datex Engstrom, Instrumentarium Corporation, Helsinki, Finland) was ensued and animals were stabilized on a volume-controlled setting with a respiratory rate (RR) of 10–12 breaths/min, a tidal volume (Vt) of 10 mL/kg/min, a positive end expiratory pressure (PEEP) of 3 cm of H_2_O, a fraction of inspired oxygen (FiO_2_) of 0.5, and an inspiratory is to expiratory (I:E) ratio of 1:2.

Anaesthesia, analgesia, and muscle relaxation were maintained with intravenous administration of midazolam (1.0 mg/kg/h) additionally supported by Isoflurane (0.5–1 %), sufentanyl citrate (4 μg/kg/h), and pancronium bromide (0.1 mg/kg/h). Limb lead electrocardiogram (ECG) and oxygen saturation (ML320/E Oximeter POD, PowerLab, AD Instruments, Oxfordshire, UK) were recorded and monitored continuously. Body temperature was maintained within normal range (37.5–38.0 °C). Intravenous infusion of Ringer’s lactate was provided. The femoral vessels (artery and vein) and pulmonary artery were catheterized to measure the mean arterial pressure, central venous pressure, and cardiac output.

### Hypercapnic phase

The hypercapnic phase was induced by resetting the ventilator to a lower Vt of 6 ml/kg/min, while maintaining other baseline parameters (RR 10–12 breaths/min, PEEP 3 cm H_2_O, FiO_2_ 0.5, and an I:E ratio of 1:2). The reduced Vt of 40 % was maintained for the remainder period of the experiment.

### CO_2_ dialysis unit set-up and cannulation procedure

**Fig. 1:**
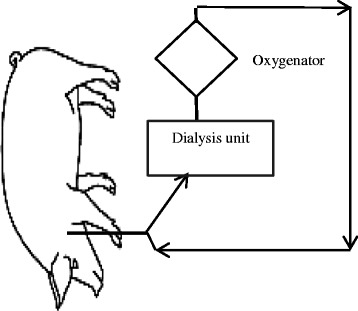
In vivo extracorporeal carbon dioxide removal study unit set-up; the hemodialyzer is replaced by diffusion membrane oxygenator (0.8 m^2^, Quadrox-iD paediatric), blood flow set at 200 mL/min, and sweep gas flow at 5 L/min (100 % O_2_)

The CO_2_ dialysis unit consisted of a continuous veno-venous hemofiltration device (Baxter BM11, Baxter Healthcare Corporation, Deerfield, IL, USA), a Bio-line coated diffusion membrane oxygenator (0.8 m^2^, Quadrox-iD paediatric, Maquet Cardiopulmonary, Rastatt, Germany) that replaced the hemoconcentrator (as shown in Fig. 1) and was primed with 200 mL of 4 % Gelofusine (B. Braun AG, Melsungen, Germany). At end of the hypercapnic phase, the animals in group 1 received an intravenous bolus injection of heparin (20 IU/kg) and the right internal jugular vein was catheterized using a regular single double lumen dialysis catheter (14 Fr. × 10”, 25 cm; Arrow international, Reading, PA, USA). A continuous infusion of heparin at the inflow port of the dialysis catheter maintained the activated partial thromboplastin time (aPTT) between 60 and 80 s, measured hourly. In group 2, the regional anticoagulation was started as soon as the catheter was placed through the inflow port of the dialysis catheter. In both groups, 100 mL of priming volume was discarded immediately after the circuit was connected to the dialysis catheter. The correct placement of the dialysis catheter was confirmed by fluoroscopy.

### Intervention phase

The sweep gas flows (100 % O_2_) through the diffusion membrane were titrated to 5 L of oxygen, and the blood flow was maintained at 200 mL/min. In group 2, Ringer’s lactate infusion was tapered accordingly, since there was continuous infusion of 4 % trisodium citrate at the inflow port of the catheter, which chelated the ionized calcium of inflowing blood. A continuous infusion of calcium gluconate 10 % at the outflow port of the catheter maintained the systemic ionized calcium. Monitoring of anticoagulation in this group was performed by hourly measurement of the ionized-calcium levels at the post-oxygenator and in the animal. The infusions of 4 % trisodium citrate and calcium gluconate were titrated accordingly to maintain the ionized-calcium level between 0.25 and 0.35 mmol/L [15] in the dialysis circuit and a systemic ionized-calcium level of 1.12–1.20 mmol/L.

### Post-intervention phase

The CO_2_ dialysis was terminated, and the dialysis circuit was disconnected after clamping the inflow and outflow ports. The animals continued to be on mechanical ventilation, while the disposable parts of the circuits were separated, flushed with saline, and visually inspected for clot formation. Parts of the diffusion membrane were collected for scanning electron microscopy (SEM) analysis, and following post-intervention phase, the animals were euthanized under general anaesthesia using a single dose of pentobarbital (150 mg/kg).

### Measurements

Hourly samples for blood gases were drawn and analysed (Gem Premier 3000, Instrumentation Laboratory, Lexington, MA, USA). Blood samples for liver function and renal function tests were collected to monitor the acute metabolic effects of citrate. The aPTT was measured using a Hemochron Jr. Signature (Edison, USA). Cardiac output was measured by thermo-dilution.

CO_2_ transfer rate was calculated using the following formula:

$$ \mathrm{C}{\mathrm{O}}_2\kern0.5em \mathrm{transfer}\ \mathrm{rate} = {Q}_{\mathrm{gas}}*\ \mathrm{Percentage}\ \mathrm{of}\ \mathrm{C}{\mathrm{O}}_2*\kern0.5em 10 $$

where *Q*_gas_ is the gas flow through the oxygenator and percentage of CO_2_ is the CO_2_ at the gas outlet port of the oxygenator, measured by capnograph (Novametrix Capnogard, Philips Respironics, Best, The Netherlands).

### Statistical analysis

Statistical analysis was performed with SPSS 21.0 software (SPSS, Chicago, IL, USA). Continuous data are presented as mean ± standard deviation or median with interquartile range as appropriate. The continuous variables were subjected to normality distribution. Accordingly, continuous variables of paired samples within the group were analysed by the Wilcoxon signed-rank test, and differences between two continuous variables were analysed by the Mann-Whitney *U* test. A *p* value <0.05 was considered statistically significant.

## Results

CO_2_ dialysis (200 mL/min) with systemic anticoagulation (group 1) and regional anticoagulation (group 2) was applied in an in vivo study. The physiological parameters (e.g. mean blood pressure, heart rate, and cardiac output) remained within normal physiological limits.

**Fig. 2:**
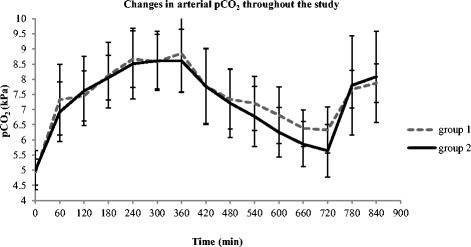
Changes in arterial pCO_2_ during CO_2_ dialysis. Values are expressed as mean±SD; Stabilisation (0-60 min); Hypercapnic phase (60-360 min); Intervention phase (360-720 min); Post-intervention phase (720-840 min); group1: heparin group; group 2: citrate group

CO_2_ transfer across the oxygenator was maximal at 45 mL/min and stabilized during the course of the intervention at 35 mL/min in both groups (*p* > 0.05). Furthermore, the mean pCO_2_ values/unit time during intervention were not significantly different between the groups that received systemic anticoagulation vs. regional anticoagulation (*p* > 0.05). A steady CO_2_ removal was observed in both groups, as depicted by the mean changes in arterial pCO_2_ (Fig. 2).

Consequently, pH improved from 7.32 ± 0.08 to 7.47 ± 0.07 in group 1 and from 7.37 ± 0.04 to 7.49 ± 0.01 in group 2, *p* < 0.05 for both groups (Table 1). Further, in group 1, a significant change in pCO_2_ was observed at the first hour of intervention and at different time points during the experimental phase. In contrast, in group 2, equilibration of CO_2_ reflected by changes in pCO_2_ occurred throughout the intervention phase (Table 2).

**Table 1:** Arterial pH levels between consecutive time points during CO_2_ dialysis

	Group 1		Group 2	
		*p* value		*p* value
End of the hypercapnic phase	7.37 ± 0.04		7.32 ± 0.08	
*t* = 60 min	7.43 ± 0.06	0.01	7.37 ± 0.08	0.02
*t* = 120 min	7.44 ± 0.05	0.03	7.45 ± 0.09	0.06
*t* = 180 min	7.45 ± 0.05	0.03	7.45 ± 0.10	0.03
*t* = 240 min	7.48 ± 0.06	0.02	7.51 ± 0.10	0.02
*t* = 300 min	7.49 ± 0.01	0.01	7.52 ± 0.10	0.02
*t* = 360 min	7.49 ± 0.01	0.01	7.47 ± 0.07	0.02

pH values are expressed as mean ± SD; group 1: heparin group; group 2: citrate group; *t*: sampling time point; *p* values are expressed for two consecutive sample points within a group during CO_2_ dialysis

**Table 2:** Arterial pCO_2_ levels between consecutive time points during CO_2_ dialysis

	Group 1		Group 2	
		*p* value		*p* value
End of the hypercapnic phase	8.9 ± 1.3		8.6 ± 1.0	
*t* = 60 min	7.8 ± 1.2	0.01	7.8 ± 1.3	0.02
*t* = 120 min	7.3 ± 0.9	0.11	7.2 ± 1.1	0.04
*t* = 180 min	7.2 ± 0.9	0.13	6.8 ± 1.0	0.02
*t* = 240 min	6.8 ± 0.9	0.01	6.2 ± 0.8	0.02
*t* = 300 min	6.4 ± 0.6	0.01	5.9 ± 0.7	0.02
*t* = 360 min	6.3 ± 0.8	0.91	5.6 ± 0.8	0.03

pCO_2_ values are expressed as mean ± SD; group 1: heparin group; group 2: citrate group; *t*: sampling time point; *p* values are expressed for two consecutive sample points within a group during CO_2_ dialysis

**Fig. 3:**
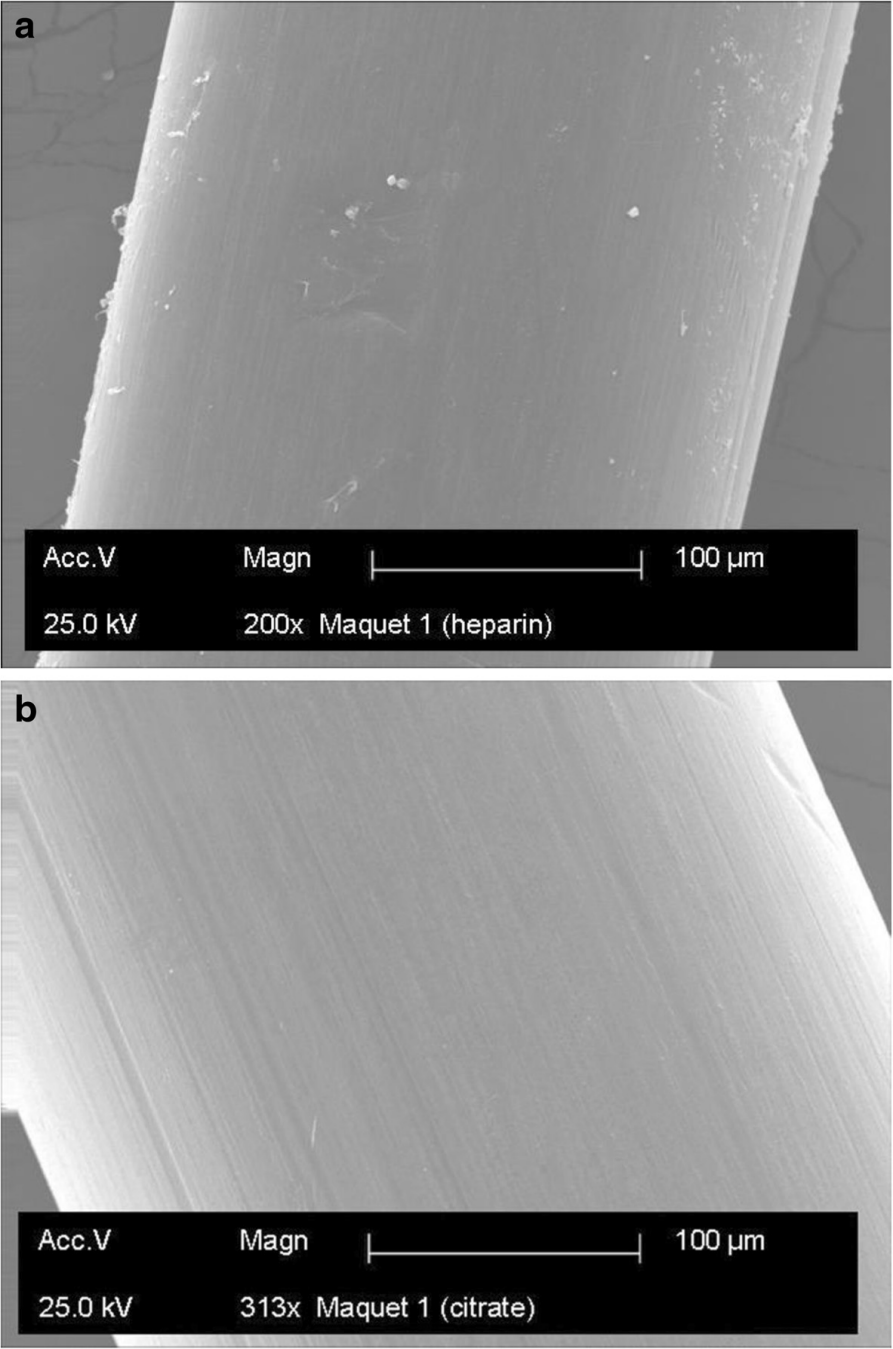
**a** Scanning electron micrograph of an oxygenator fibre in the heparin group showing some cellular and fibrin adhesion. **b** Scanning electron micrograph of an oxygenator fibre in the citrate group

The arterial pO_2_ during intervention remained at a mean of 13.2 ± 0.3 kPa in group 1 while it was a mean of 14.1 ± 0.7 kPa in group 2. Transoxygenator pressure difference varied between 10 and 15 mmHg. The liver function tests (alkaline phosphatase 88.0 ± 11.0 IU/L; gamma glutaryl transferase 14 ± 2.0 IU/L; aspartate aminotransferase 22.0 ± 5.0 IU/L; alanine aminotransferase 44.0 ± 2.0 IU/L; total bilirubin 2.2 ± 0.2 μmol/L; direct bilirubin 2.2 ± 0.2 μmol/L) and renal function test (creatinine103.0 ± 0.7 μmol/L) were within normal range. SEM analysis (Fig. 3a) of the diffusion membrane revealed only some cellular and fibrin adhesion on the oxygenator fibre in the heparin group (Fig. 3b).

## Discussion

Low-flow CO_2_ removal was efficient in establishing eucapnia in a swine model. A mean CO_2_ extraction of 40 mL/min through a modified renal dialysis unit permitted adequate gas transfer under constant low volume ventilatory support. Consequently, arterial pH improved within the initial hour of intervention, highlighting the efficacy of the gas-exchange diffusion membrane even at a low blood flow rate.

Hypercapnic respiratory failure worsens prognosis and increases mortality in COPD patients [18, 19]. The pathophysiological alterations precipitating this condition include dynamic hyperinflation and expiratory flow limitation as a result of respiratory muscle fatigue, which further aggravates respiratory acidosis. NIV is therefore considered as a standard mode of treatment to provide ventilation support among these patients, to improve acidemia (pH ≤7.35 and/or arterial pCO_2_ ≥6.0 kPa [1]. In the majority of patients, NIV application has been found effective to avert imminent respiratory arrest. However, a well-demarcated contradiction to apply NIV is the presence of co-morbidities [20]. Moreover, failure of NIV hinders its application or even the effectiveness of the therapy. Consequently, an invasive approach to provide ventilation is applied, which is associated with a poor survival rate [21]. Therefore, a minimally invasive form of support through extracorporeal CO_2_ removal technology has been increasingly considered as a viable alternative to standard therapies [3].

In the recent years, application of ECCO_2_R devices has been gathering interest to support patients in hypercapnic respiratory failure refractory to standard therapy [3, 8]. As application of ECCO_2_R has been demonstrated to be effective, using blood flows of about 400–1000 mL/min supports gas transfer and ameliorates moderate to severe hypercapnic acidosis [8, 22], thereby reducing pCO_2_ (from 10.5 to 8.7 kPa) and improving pH (from 7.28 ± 0.08 to 7.34 ± 0.07) following 1 h of support at a blood flow rate of approximately 500 mL/min [8]. Recently, Kargiannidis et al. reported a swine study which showed improvement of pCO_2_ (from about 14.4 to 7.5 kPa) with acidemia equilibrating from 7.13 ± 0.08 to 7.41 ± 0.07 within 15 min of ECCO_2_R support at a blood flow rate of 1000 mL/min [22]. Albeit, one of the largely unrecognized concern prevails among such patients when hypercapnia is optimized too rapidly, resulting in metabolic alkalosis which can increase the duration of recovery and hospital stay [23]. Moreover, in situations of acute-on-chronic hypercapnic respiratory failure, a steady removal of CO_2_ might be necessary for an extensive period of time, as CO_2_ persists in HCO_3_-form in slow compartments [22]. Extensive periods of high-flow ECCO_2_R support might prove counter-productive, as we might plausibly alter the acid-base balance dramatically. Therefore, low-flow CO_2_ removal would be a more controlled and physiological alteration to establish homeostasis in a chronically altered state. As described in our study, a commonly used renal dialysis unit was adapted by incorporating a diffusion membrane oxygenator replacing the hemodialyzer. CO_2_ dialysis in our observation resulted in a lower CO_2_ extraction (approximately 40 mL/min) compared to currently available veno-venous pump-driven devices (approximately 80 mL/min) [9, 11]. Although, CO_2_ dialysis in this current study seems to have a lower CO_2_ extraction rate, the ability to increase blood flows with prevalent dialysis devices provides the possibility to increase higher amount of CO_2_ extraction when indicated, while opting for a larger size cannula. In patients with underlying renal insufficiency and acute respiratory distress syndrome, Forster and colleagues described the role of low-flow CO_2_ removal, where the introduction of a hollow-fibre oxygenator in a renal replacement unit ameliorated acidosis and improved pCO_2_ [24]. Although this auxiliary support facilitated improvements, the risk of anticoagulation failure leading to clot formation or bleeding with systemic anticoagulation remains a challenge.

In general, an extracorporeal device such as CO_2_ removal system requires systemic anticoagulation [25], to maintain patency and reduce thromboembolic complications. To date, heparin remains the most commonly used anticoagulant in these devices, potentially increasing the risk of bleeding. Additionally, the use of unfractionated heparin in patients susceptible for bleeding can induce thrombocytopenia and hence propagate the risk of systemic bleeding [12, 26]. It would be, therefore, attractive to implement regional anticoagulation with citrate as practised widely in renal dialysis [15] to reduce the potential risk of bleeding. The practice of regional anticoagulation in CO_2_ removal has not been prevalent. Cardenas et al. described the use of regional citrate anticoagulation in a CO_2_ removal system, but they were challenged with the maintenance of adequate systemic calcium levels [27]. In our study, we used a standard protocol for regional citrate anticoagulation, where citrate was infused into the inlet port of the catheter chelating calcium. The chelated calcium inhibited the activation of calcium-dependent coagulation factors in the dialysis circuit, providing optimum anticoagulation. The infusion of calcium gluconate in the outlet port of the catheter reversed the citrate effect.

The use of regional anticoagulation with citrate also has additional benefits, such as improving the circuit survival time [15]. Moreover, the secondary gain in administering trisodium citrate is the formation of sodium bicarbonate, which is an end-product of citrate metabolism occurring in the liver, skeletal muscle, and kidney [17]; this might buffer the excess acid. However, this beneficial chemical buffering of CO_2_ was observed less evidently, conceivably in view of the efficient CO_2_ dialysis system.

Gross thrombus formation, a potential complication in low-flow conditions, was not observed in either the heparin group or the citrate group. Although SEM analysis from the heparin group revealed only some cellular and fibrin adhesion over the diffusion membrane, similar observations were not prominent from the analysis in the citrate group. This latter observation might be due to the chelation of calcium and subsequent decrease in the inflammatory reactions that reduce the activation of leukocytes and monocytes [28], leading to less adhesion.

We recognized some limitations with this study. First, the use of healthy animals did not allow us to completely mimic the acute-on-chronic hypercapnic respiratory failure, where severe airflow limitation could not be simulated. Additionally, this limitation also prevents us to understand what could potentially challenge in clinical practice if there were to be device failure in non-ventilated patients. Furthermore, increasing acidosis (pH <7.25) over an extended period could not be replicated, due to a potential source of arrhythmia and, to an extent, stabilization of hypercapnic levels during the hypercapnic phase of the study. Nevertheless, the steady and gradual improvement in arterial pCO_2_ and pH utilizing CO_2_ dialysis in the given circumstance proved effective in ameliorating acute hypercapnia irrespective of the nature of anticoagulation. In the event of prolonged support, the influence of regional anticoagulation on CO_2_ dialysis circuit life is worth investigating.

## Conclusions

In conclusion, we demonstrated that low-flow CO_2_ dialysis using a modified renal replacement system improved arterial pCO_2_ significantly, thereby optimizing pH. Furthermore, regional anticoagulation with citrate would be an alternative option to circumvent bleeding complications. However, further studies are needed to determine if this approach is a viable option to potentially support patients with acute-on-chronic respiratory failure.

## References

[1] Global Strategy for the Diagnosis MaPoC, Global Initiative for Chronic Obstructive Lung Disease (GOLD) (2014) Available from: http://www.goldcopd.org/guidelines-global-strategy-for-diagnosis-management.html

[2] Hoo GW, Hakimian N, Santiago SM (2000). Hypercapnic respiratory failure in COPD patients: response to therapy. Chest.

[3] Terragni P, Maiolo G, Ranieri VM (2012). Role and potentials of low-flow CO(2) removal system in mechanical ventilation. Curr Opin Crit Care.

[4] Gattinoni L, Pesenti A, Kolobow T, Damia G (1983). A new look at therapy of the adult respiratory distress syndrome: motionless lungs. Int Anesthesiol Clin.

[5] Schmidt M, Tachon G, Devillers C, Muller G, Hekimian G, Brechot N, Merceron S, Luyt CE, Trouillet JL, Chastre J, Leprince P, Combes A (2013). Blood oxygenation and decarboxylation determinants during venovenous ECMO for respiratory failure in adults. Intensive Care Med.

[6] Berdajs DA, de Stefano E, Delay D, Ferrari E, Horisberger J, Ditmar Q, von Segesser LK (2011). The new advanced membrane gas exchanger. Interact Cardiovasc Thorac Surg.

[7] Khoshbin E, Westrope C, Pooboni S, Machin D, Killer H, Peek GJ, Sosnowski AW, Firmin RK (2005). Performance of polymethyl pentene oxygenators for neonatal extracorporeal membrane oxygenation: a comparison with silicone membrane oxygenators. Perfusion.

[8] Burki NK, Mani RK, Herth FJ, Schmidt W, Teschler H, Bonin F, Becker H, Randerath WJ, Stieglitz S, Hagmeyer L, Priegnitz C, Pfeifer M, Blaas SH, Putensen C, Theuerkauf N, Quintel M, Moerer O (2013). A novel extracorporeal CO(2) removal system: results of a pilot study of hypercapnic respiratory failure in patients with COPD. Chest.

[9] Batchinsky AI, Jordan BS, Regn D, Necsoiu C, Federspiel WJ, Morris MJ, Cancio LC (2011). Respiratory dialysis: reduction in dependence on mechanical ventilation by venovenous extracorporeal CO2 removal. Crit Care Med.

[10] Iacovazzi M, Oreste N, Sardelli P, Barrettara B, Grasso S (2012). Extracorporeal carbon dioxyde removal for additional pulmonary resection after pneumonectomy. Minerva Anestesiol.

[11] Livigni S, Maio M, Ferretti E, Longobardo A, Potenza R, Rivalta L, Selvaggi P, Vergano M, Bertolini G (2006). Efficacy and safety of a low-flow veno-venous carbon dioxide removal device: results of an experimental study in adult sheep. Crit Care (London, England).

[12] Gray BW, Haft JW, Hirsch JC, Annich GM, Hirschl RB, Bartlett RH (2015). Extracorporeal life support: experience with 2,000 patients. Am Soc Artif Internal Organ.

[13] Extracorporeal Life Support Organization (2015) ELSO registry. Available at www.elso.org/Registry/Statistics/InternationalSummary.aspx

[14] Wu MY, Hsu YH, Bai CH, Lin YF, Wu CH, Tam KW (2012). Regional citrate versus heparin anticoagulation for continuous renal replacement therapy: a meta-analysis of randomized controlled trials. Ame J Kidney Dis.

[15] Bagshaw SM, Laupland KB, Boiteau PJ, Godinez-Luna T (2005). Is regional citrate superior to systemic heparin anticoagulation for continuous renal replacement therapy? A prospective observational study in an adult regional critical care system. J Crit Care.

[16] Morabito S, Pistolesi V, Tritapepe L, Vitaliano E, Zeppilli L, Polistena F, Fiaccadori E, Pierucci A (2013). Continuous venovenous hemodiafiltration with a low citrate dose regional anticoagulation protocol and a phosphate-containing solution: effects on acid-base status and phosphate supplementation needs. BMC Nephrol.

[17] Mariano F, Bergamo D, Gangemi EN, Hollo Z, Stella M, Triolo G (2011). Citrate anticoagulation for continuous renal replacement therapy in critically ill patients: success and limits. Int J Nephrol.

[18] Connors AF, Dawson NV, Thomas C, Harrell FE, Desbiens N, Fulkerson WJ, Kussin P, Bellamy P, Goldman L, Knaus WA (1996). Outcomes following acute exacerbation of severe chronic obstructive lung disease. The SUPPORT investigators (Study to Understand Prognoses and Preferences for Outcomes and Risks of Treatments). Am J Respir Crit Care Med.

[19] Hoogendoorn M, Hoogenveen RT, Rutten-van Molken MP, Vestbo J, Feenstra TL (2011). Case fatality of COPD exacerbations: a meta-analysis and statistical modelling approach. Eur Respir J.

[20] Ambrosino N, Vagheggini G (2008). Noninvasive positive pressure ventilation in the acute care setting: where are we?. Eur Respir J.

[21] Menzies R, Gibbons W, Goldberg P (1989). Determinants of weaning and survival among patients with COPD who require mechanical ventilation for acute respiratory failure. Chest.

[22] Karagiannidis C, Kampe KA, Sipmann FS, Larsson A, Hedenstierna G, Windisch W, Mueller T (2014). Veno-venous extracorporeal CO2 removal for the treatment of severe respiratory acidosis: pathophysiological and technical considerations. Crit Care (London, England).

[23] Banga A, Khilnani GC (2009). Post-hypercapnic alkalosis is associated with ventilator dependence and increased ICU stay. COPD.

[24] Forster C, Schriewer J, John S, Eckardt KU, Willam C (2013). Low-flow CO_2_ removal integrated into a renal-replacement circuit can reduce acidosis and decrease vasopressor requirements. Crit Care.

[25] Conrad SA, Zwischenberger JB, Grier LR, Alpard SK, Bidani A (2001). Total extracorporeal arteriovenous carbon dioxide removal in acute respiratory failure: a phase I clinical study. Intensive Care Med.

[26] Tolwani A, Wille KM (2012). Advances in continuous renal replacement therapy: citrate anticoagulation update. Blood Purif.

[27] Cardenas VJ, Miller L, Lynch JE, Anderson MJ, Zwischenberger JB (2006). Percutaneous venovenous CO_2_ removal with regional anticoagulation in an ovine model. Am Soc Artificial Internal Organ.

[28] Bos JC, Grooteman MP, van Houte AJ, Schoorl M, van Limbeek J, Nube MJ (1997). Low polymorphonuclear cell degranulation during citrate anticoagulation: a comparison between citrate and heparin dialysis. Nephrol Dial Transplant.

